# G6PD promotes renal cell carcinoma proliferation through positive feedback regulation of p-STAT3

**DOI:** 10.18632/oncotarget.22566

**Published:** 2017-11-20

**Authors:** Qiao Zhang, Zhe Yang, Qiaoqiao Han, Honggang Bai, Yanling Wang, Xiaojia Yi, Zihan Yi, Lijuan Yang, Lu Jiang, Xin Song, Yingmin Kuang, Yuechun Zhu

**Affiliations:** ^1^ Department of Biochemistry and Molecular Biology, School of Basic Medical Sciences, Kunming Medical University, Chenggong District, Kunming 650500, Yunnan Province, China; ^2^ Department of Pathology, The First Affiliated Hospital of Kunming Medical University, Wuhua District, Kunming 650032, Yunnan Province, China; ^3^ Department of Pathology, The Second Affiliated Hospital of Kunming Medical University, Wuhua District, Kunming 650101, Yunnan Province, China; ^4^ Department of Cancer Biotherapy Center, The Third Affiliated Hospital of Kunming Medical University, Xishan District, Kunming 650118, Yunnan Province, China; ^5^ Department of Organ Transplantation, The First Affiliated Hospital of Kunming Medical University, Wuhua District, Kunming 650032, Yunnan Province, China

**Keywords:** G6PD, renal cell carcinoma, proliferation, p-STAT3, positive feedback regulation

## Abstract

Ectopic Glucose 6-phosphate dehydrogenase (G6PD) expression plays important role in tumor cell metabolic reprogramming and results in poor prognosis of multiple malignancies. Our previous study indicated that G6PD is overexpressed in clear cell renal cell carcinoma (ccRCC), the most common subtype of RCC. However, its role in RCC is still unclear. Here, we demonstrate that G6PD is not only up-regulated in all types of RCC specimens but also displays higher activities in RCC cell lines. G6PD overexpression promoted RCC cell proliferation, altered cell cycle distribution, and enhanced xenografted RCC development. G6PD up-regulated ROS generation by facilitating NADPH-dependent NOX4 activation, which led to increased expression of p-STAT3 and CyclinD1. Enhanced ROS generation rescued the p-STAT3 and CyclinD1 expression reduction in G6PD-knockdown cells, while ROS scavengers reversed the up-regulated p-STAT3 and CyclinD1 expression in G6PD-overexpressing cells. Furthermore, p-STAT3 activated G6PD gene expression via binding to the G6PD promoter, demonstrating that p-STAT3 forms a positive feedback regulatory loop for G6PD overexpression. G6PD expression was up or down-regulated in response to the impact of p-STAT3 activators or inhibitors. Therefore, G6PD may be an effective RCC therapeutic target.

## INTRODUCTION

Glucose 6-phosphate dehydrogenase (G6PD), the first and rate-limiting enzyme of the pentose phosphate pathway (PPP), plays critical roles for nucleotide precursors generation and redox homeostasis maintenance [1]. Lately, increasing evidences show that high expression of G6PD predicts poor overall survival of patients with numbers of cancers, indicating that G6PD may play important roles in tumorigenesis [1, 2]. In addition to the so-called Warburg effect, G6PD was aberrantly activated in order to generate sufficient building blocks required for rapid proliferation and adaptation of cancer cells to the altered internal and external environment [1, 3]. Our recent studies demonstrate that G6PD is significantly higher expressed in advanced status of clear cell renal cell carcinoma (ccRCC) and closely correlates to the tumor extent, lymph node metastasis, Fuhrman grade, TNM stage and poor overall survival of ccRCC [4]. ccRCC is the most common subtypes of RCC and constitutes approximately 80∼90% of RCCs. However, the expression pattern of G6PD in all the RCC cases is poorly reported, and the roles and underlying mechanisms of G6PD in RCC development, to date, remain largely unknown [4].

RCC is the most prevalent and dangerous malignancy of the kidney with a median survival time of about 13 months and less than 10% of patients with RCC will survive more than 5 years [5–7]. Each year, approximately 210,000 new cases of RCC are diagnosed [5, 6]. Although many advances have been made in RCC diagnosis and treatment, the molecular mechanisms that govern RCC initiation and progression are poorly understood. Therefore, identifying novel genes that are functionally involved in tumorigenesis is critical and may provide more sophisticated early diagnostic and therapeutic strategies for RCC.

Many studies have suggested that RCC is fundamentally a metabolic disease [8] and that an altered metabolism is involved in RCC tumorigenesis [9, 10]. Moreover, numerous genes involved in RCC development play an essential part in mediating carcinoma cell metabolism [11, 12]. Based on these facts, we tentatively hypothesize that there must be a close underlying correlation between G6PD and RCC tumorigenesis. It has been reported that, G6PD plays important roles in the regulation of reactive oxygen species (ROS) generation [1]. As the PPP is activated, G6PD is usually considered the pacesetter for nicotinamide adenine dinucleotide phosphate (NADPH) production in cancer cells [1, 2, 13]. NADPH is a substrate of NADPH oxidases and functionally important hydrogen donor that serves as a cofactor in anabolic reactions of fatty acids, isoprenoids, and aromatic amino acids synthesis [14]. ROS are primarily produced by NADPH oxidases (NOX) of the NOX family which includes five homologs, NOX1 to NOX5, and two related enzymes DUOX1 and DUOX2 [15–17]. It has been confirmed that NOX oxidases, particularly the isoform NOX4, are the major source of ROS in RCC [18–20].

NOX4, especially the membrane-bound NOX4, plays a major role in regulating cellular functions and is thought to be an electron transporter [13, 15, 21]. In combination with NADPH, NOX4 could pass the electrons to an electron acceptor according to the equation NADPH→FAD→Heme→O_2_ to generate O_2_^-.^. Then, O_2_^-.^ will be catalyzed sequentially into the downstream products containing H_2_O_2_ and OH^.^. O_2_^-^, H_2_O_2_ and OH^.^ are members of the ROS family [15]. Accordingly, both G6PD and NOX4 are important in cellular ROS metabolism and may synergistically act in regulation of redox homeostasis in RCC [13]. However, limited information is available about the regulatory relationship between G6PD and ROS levels in RCC. Before the experiment carried out, we could not assume that G6PD overexpression will increase or decrease ROS generation. Because it has been clarified that G6PD could probably promote ROS production through the process we described, but may also have the abilities to eliminate ROS via the reduced form of glutathione. Reduced glutathione (GSH) depends on NADPH to maintain its reducing state and acts as a scavenger for dangerous oxidative metabolites [22].

In this study, we firstly analyzed the expression profile of G6PD using bioinformatics and immunohistochemical techniques. To better understand the function of G6PD in RCC, stable G6PD-overexpressing and knockdown RCC cells were established, and the effects of G6PD on RCC proliferation were assessed both *in vitro* and *in vivo*. Furthermore, the potential molecular mechanisms underlying G6PD-promoted tumorigenesis and the reason for aberrant G6PD expression in RCC were investigated.

## RESULTS

### G6PD is overexpressed in human renal cell carcinoma samples

**Figure 1:**
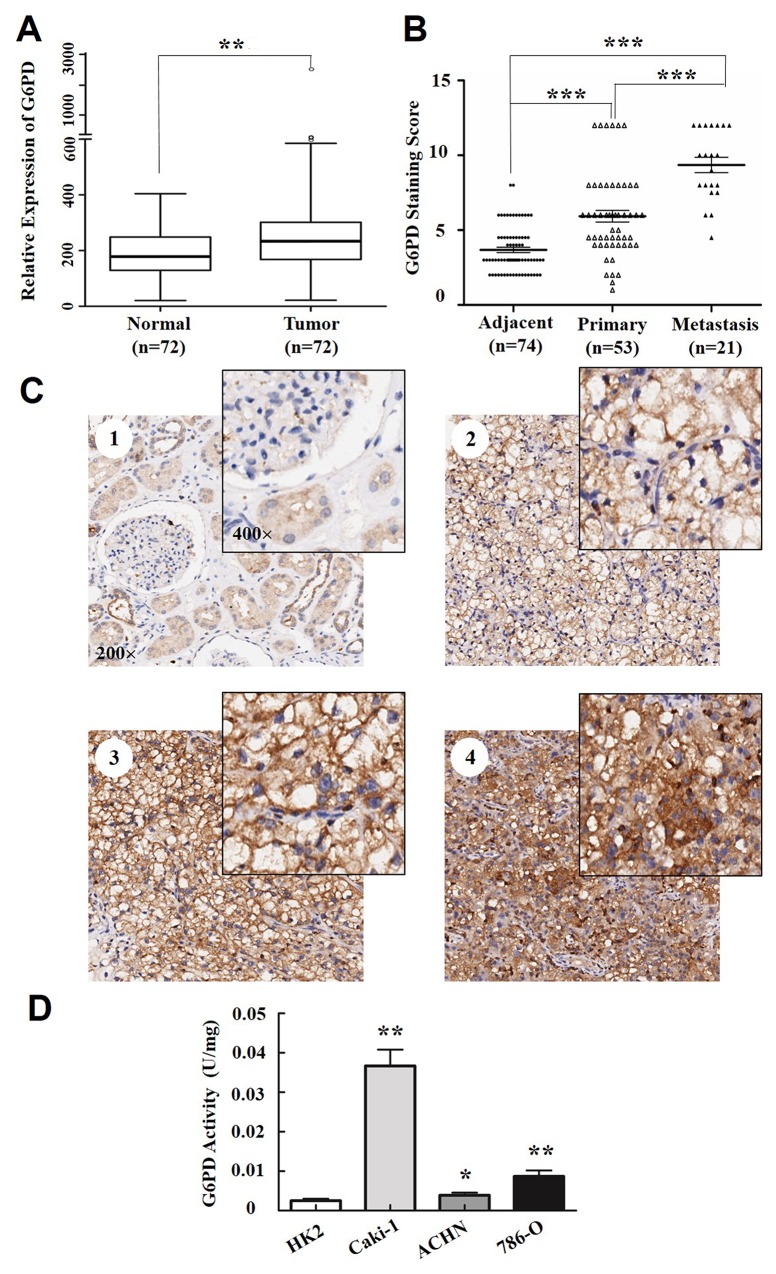
G6PD is overexpressed in RCC **(A)** Expression profiling of G6PD from Gene Expression Omnibus (GEO) datasets in ccRCC samples and normal renal tissues (n=72). ^**^ *p* < 0.01 vs. Normal (Wilcoxon rank-sum test). **(B)** Staining scores of G6PD in adjacent normal tissues (n=74), primary RCC without lymph node or distant metastasis (n=53) and metastasis RCC (n=21). ^***^ *p* < 0.001 vs. Adjacent or Primary (Kruskal-Wallis one-way analysis). **(C)** Representative images of immunohistochemical staining and cellular distributions for G6PD in noncancerous renal tissues (**C1**, weak G6PD expression), early TNM stage (**C2**, moderate G6PD expression), Stage III and Stage IV (**C3-C4**, strong G6PD expression) RCC samples. Images were captured using 20× and 40× objective lens. **(D)** G6PD activity assays in HK2 (human renal tubular epithelial cell line) and 3 RCC cell lines (Caki-1, ACHN and 786-O). ^*^ *p* < 0.05, ^**^ *p* < 0.01 vs. HK2 (one-way ANOVA). Values are means ± SD of three independent experiments, each performed in triplicate.

A gene expression profile reveals essential clues about the function of a gene. We previously reported the aberrant expression of G6PD in ccRCC [4]. In the present study, we further explore the association between G6PD and RCC through data mining of the public Gene Expression Omnibus (GEO) of 72 ccRCC and the adjacent non-tumor tissues (GSE53757). The results showed that G6PD mRNA levels were significantly higher in ccRCC tissues than in the adjacent tissues (*p* < 0.01, Figure 1A). This conclusion is not totally the same as our previous statistical analyses of The Cancer Genome Atlas (TCGA) datasets [4], but provides sufficient information for further unravelling the correlation between G6PD overexpression and RCC tumor initiation and progression.

RCC is a type of malignant tumor originating from the epithelial cells of the renal tubule or collecting duct in the kidney. The most predominant subtype of RCC is ccRCC and the other histologic subtypes of RCC, papillary (pRCC) and chromophobe (chRCC) constitute 15% and 5% of RCC cases, respectively [23]. To examine the pathological relevance of G6PD in all RCCs development, the protein levels and cellular distribution of G6PD in RCC (60 ccRCC, 10 pRCC and 4 chRCC samples which were in parallel with the proportion of each RCC subtype) were analyzed using immunohistochemistry. Though there were no obvious expression differences between the different subtypes, the results have showed that the expression of G6PD was significantly increased in the total of 74 RCC specimens (*p* < 0.001, Table 1). High expression level of G6PD was detected in 18.92% (14/74) of the non-cancerous renal tissues but in 67.57% (50/74) of the RCC tissues. Moreover, G6PD expression was significantly higher in the RCC metastasis than that detected in normal adjacent tissues or primary RCC without lymph node or distant metastasis (Figure 1B). As shown in Figure 1C1, the predominant G6PD localization within the normal parenchyma was in renal tubular cells, but at lower expression levels in other cell types, including glomerular mesangial cells. Additionally, G6PD was mainly localized in the cytoplasm of the renal tumor cells, with different staining intensities in different TNM stages of RCC (Figure 1C2-C4).

**Table 1:** Expression of G6PD in human renal cell carcinoma (RCC)

Samples	No.	Expression of G6PD			*p* value
		Low (%)	High (%)	Avg. score	
Adjacent	74	60 (81.08)	14 (18.92)	3.682	< 0.001
RCC	74	24 (32.43)	50 (67.57)	6.905	

*χ*^2^ test

Likewise, the enzyme activities of G6PD were also significantly higher in RCC cell lines (ACHN, 786-O, Caki-1) than in the renal tubular epithelial cell line HK2 (Figure 1D), according to the results from analyses using the G6PD assay kit. These results indicate that G6PD is highly expressed and overactive in RCC and might be tightly correlated to renal tumorigenesis.

### G6PD promotes RCC cell proliferation *in vitro* and enhances tumor growth *in vivo*

To explore the function of G6PD overexpression in RCC, we firstly transfected ACHN and 786-O cells with pBABE-puro-G6PD, or pSR-GFP/Neo-G6PD shRNA plasmid to establish stable G6PD-overexpressing or knockdown RCC cell lines. Real-time RT-PCR and Western blot were used to quantify the expression levels of G6PD. The results showed that these stable cell lines were established successfully (Supplementary Figure 1).

**Figure 2:**
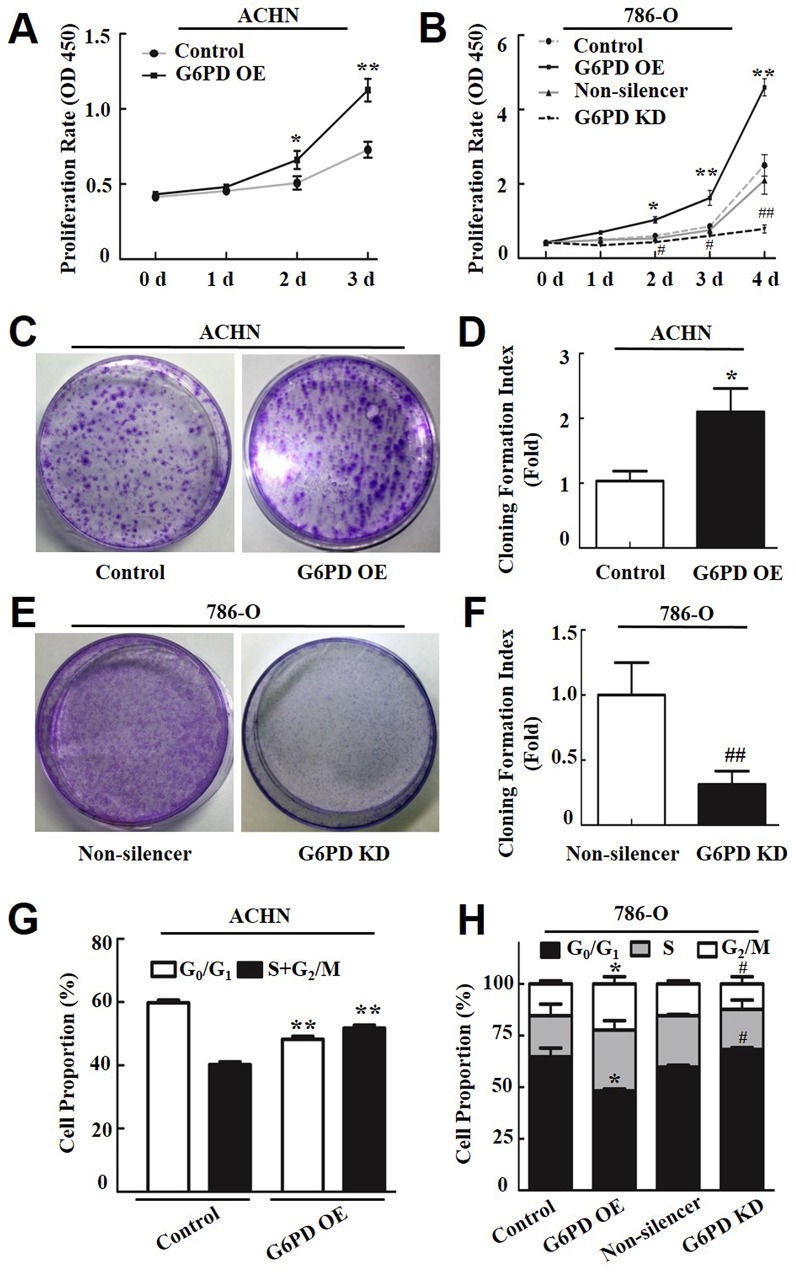
G6PD enhances RCC cell proliferation and affects cell-cycle phase distribution **(A-B)** The cell proliferation rate of transfected ACHN (A) and 786-O cells (B) was determined using the CCK-8 assay at indicated days post-seeding. The data represent three independent experiments, each performed in triplicate. **(C-F)** The plate clone formation assay and quantification assessment were performed to determine the proliferation rate of ACHN (C-D) and 786-O (E-F) cells transfected with the G6PD OE plasmid, G6PD KD plasmid, or the control vectors. **(G-H)** Flow cytometry analysis of ACHN (G) and 786-O (H) cell cycle distributions. The data are means ± SD from three independent experiments, each performed in triplicate. ^*^ *p <* 0.05, ^**^ *p <* 0.01 vs. Control; ^#^ *p <* 0.05, ^##^ *p* < 0.01 vs. Non-silencer (unpaired Student *t*-test). Control, G6PD OE, Non-silencer and G6PD KD represent cells transfected with pBABE-puro, pBABE-puro-G6PD, pSR-GFP/Neo-Non-silencer and pSR-GFP/Neo-G6PD shRNA plasmids, respectively.

Based on the importance of G6PD in nucleotide precursors production, we wonder whether G6PD overexpression plays a role in promoting RCC cell growth. As expected, the Cell Counting Kit-8 (CCK-8) detection results showed that when G6PD protein level was up or down-regulated, notable promotion or reduction of cell proliferation were observed in ACHN (Figure 2A) and 786-O stable cell lines (Figure 2B). Consistently, the colony formation assay also revealed that G6PD overexpression in ACHN cells showed significantly increased colony formation index compared with the control cells (Figure 2C, 2D), while reduced colony formation index were observed in G6PD-knockdown 786-O cells (Figure 2E, 2F).

To further elucidate the role of G6PD underlying RCC cell growth, the cell cycle distribution of RCC cells was analyzed by flow cytometry. The results revealed that G6PD-overexpressing ACHN and 786-O cells had a decreased cell population in the G_0_/G_1_ phase and a significant increase in the S and G_2_/M phase compared with the control cells (Figure 2G, 2H). Meanwhile, G6PD knockdown increased the proportion of 786-O cells in the G_0_/G_1_ phase and decreased the proportion of cells in the S and G_2_/M phases (Figure 2H). These results indicate that G6PD facilitates the cell cycle and promotes RCC cell proliferation.

**Figure 3:**
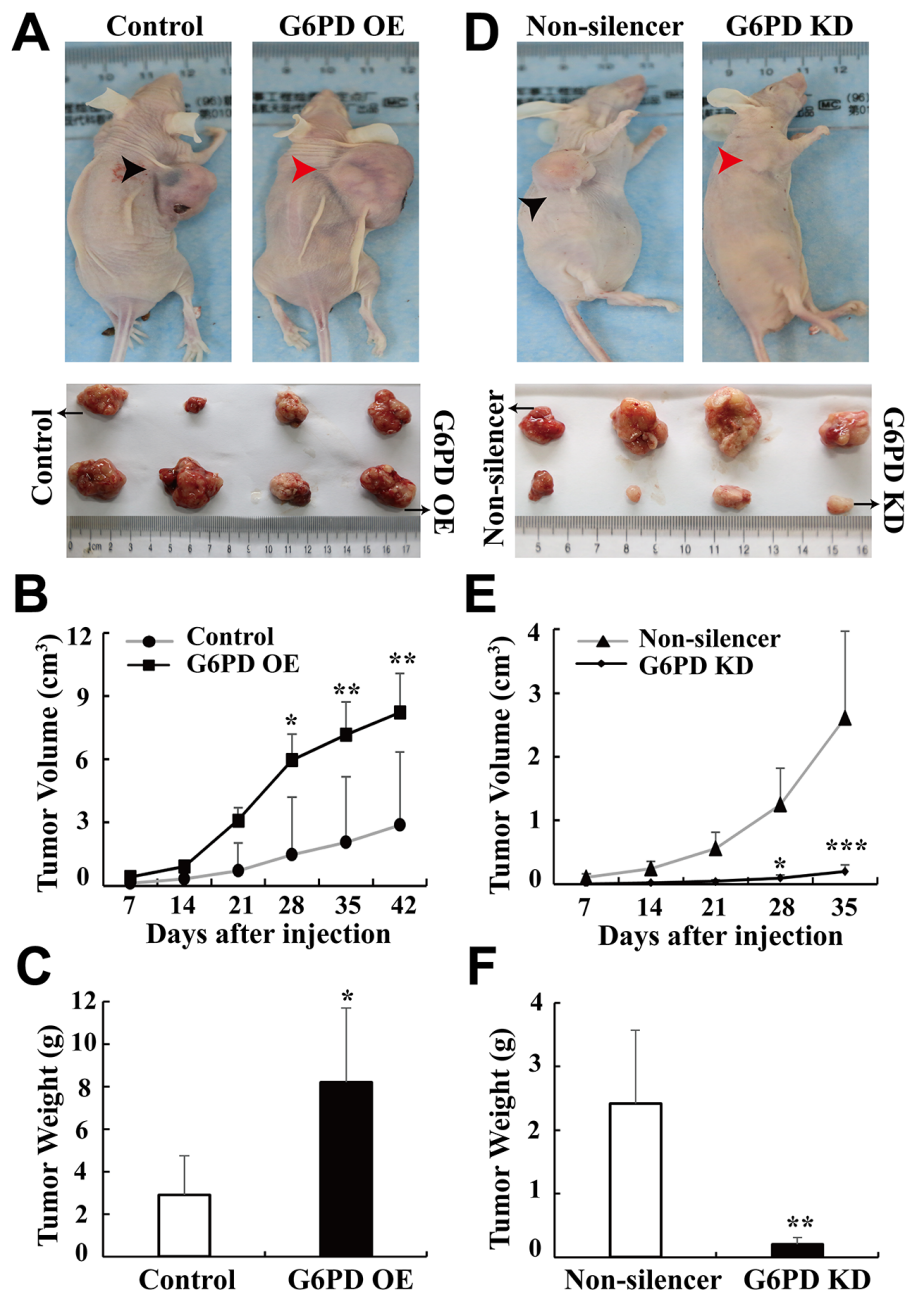
G6PD promotes xenografted RCC development *in vivo* **(A-C)** 786-O cells stably transfected with pBABE-puro (Control) or pBABE-puro-G6PD (G6PD OE), **(D-E)** 786-O cells stably transfected with pSR-GFP/Neo-Non-silencer (Non-silencer) or pSR-GFP/Neo-G6PD shRNA (G6PD KD). 1 × 10^7^ appropriate cells were resuspended in 200 μl PBS and injected subcutaneously into the right oxter flank of each BALB/c nude mice. Representative images of tumor-bearing mice (top panel) and the tumors isolated from each group (bottom panel) at day 42 (Control or G6PD OE) or day 35 (Non-silencer or G6PD KD) are shown in A and D. Tumor size was monitored at indicated days post-injection and the results are shown in B and E. ^*^ *p* < 0.05, ^**^ *p* < 0.01, ^***^ *p* < 0.001 vs. Control or Non-silencer (two-way ANOVA). Tumor weights in each group were measured after the mice were sacrificed and samples harvested **(C, F)**. ^*^ *p* < 0.05, ^**^ *p* < 0.01 vs. Control or Non-silencer (unpaired Student *t*-test).

The above *in vitro* results demonstrate that G6PD may play an oncogenic role in RCC. Therefore, we subsequently used xenograft models in nude mice to investigate whether G6PD promotes RCC tumor growth *in vivo*. G6PD-overexpressing, G6PD-knockdown or respective control 786-O cells were injected subcutaneously into the right oxter flanks of nude mice. Tumor volume was measured on the indicated days after injection. We found that the growth of tumor and tumor size were significantly faster and larger with G6PD-overexpressing cells compared with the control cells (Figure 3A-3C). Conversely, the G6PD-knockdown cells produced slower and smaller tumors than those with the Non-silencer cells (Figure 3D-3F). These results confirm that G6PD potentiates RCC tumorigenesis *in vivo*.

Taken together, *in vitro* and *in vivo* results indicate that G6PD overexpression increases RCC cell proliferation and enhances RCC tumorigenesis, whereas G6PD silencing reduces RCC cells growth and inhibits xenograft development.

### G6PD stimulates ROS production via the up-regulation of NOX4 activity

**Figure 4:**
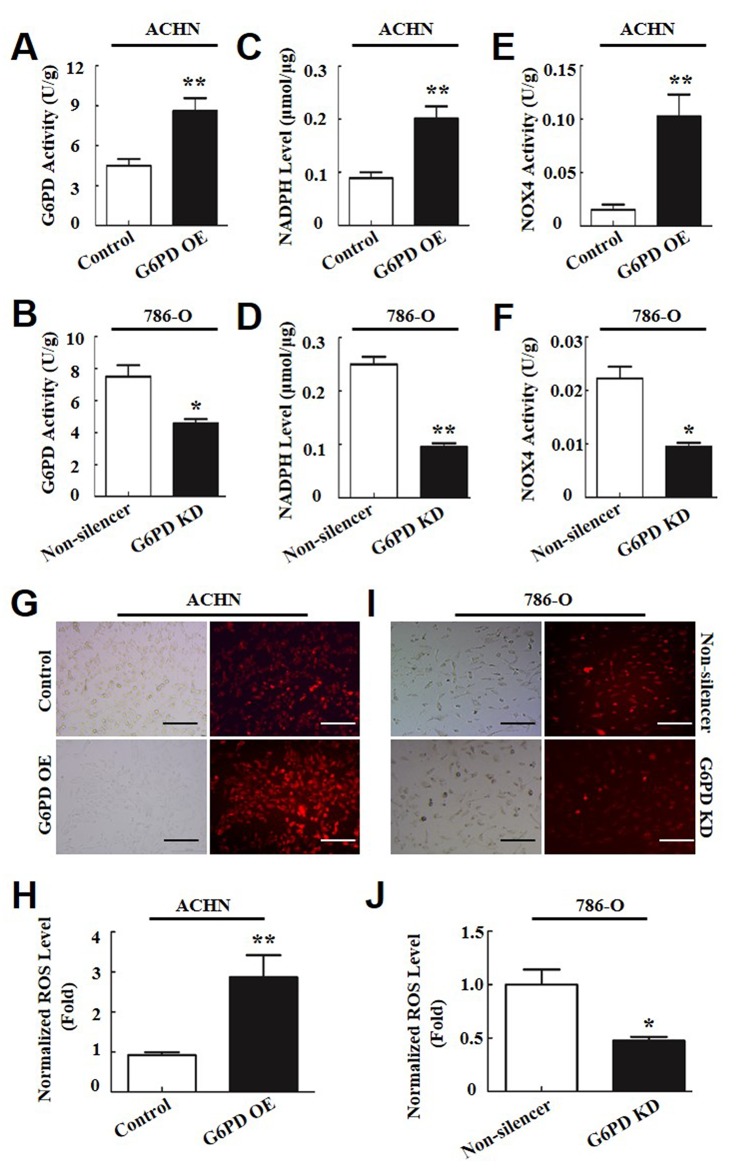
G6PD promotes ROS production by increasing NOX4 activity in RCC cells **(A-F)** G6PD activities (A-B), NADPH levels (C-D) and NOX4 activities (E-F) were analyzed using the respective assay kit in ACHN (A, C, E) and 786-O cells (B, D, F). **(G-J)** ROS accumulation was measured using fluorescence microscopy (G, I) and flow cytometry analysis (H, J) in ACHN (G, H) and 786-O **(I, J)** cells. The left images of G and I were taken under normal light and the right images were taken under fluorescence. Scale bar = 200 μm. The data are means ± SD from three independent experiments, each performed in triplicate. ^*^ *p* < 0.05, ^**^ *p* < 0.01, ^***^ *p* < 0.001 vs. Control or Non-silencer (unpaired Student *t*-test).

As the key enzyme of the pentose phosphate pathway, G6PD plays an important role in the maintenance of the cellular redox balance by cooperating with NADPH oxidase 4 (NOX4) and synergistically regulating the production of reactive oxygen species (ROS), which is often closely correlated with tumor initiation and development [1, 24]. To explore the mechanism of G6PD-regulated RCC cell proliferation, the activities of G6PD and NOX4, and the NADPH and ROS levels of RCC cells, were measured. As shown in Figure 4A, 4B, 786-O cells revealed a significantly high basal activity of G6PD (7 ± 0.4 U/g) in comparison with the ACHN cells (4 ± 0.4 U/g). Moreover, with G6PD expression levels up or down-regulated, notable promotion or inhibition of G6PD activities were observed in corresponding ACHN (Figure 4A) or 786-O (Figure 4B) cells. Additional results indicated that overexpression of G6PD increased the levels of NADPH (Figure 4C), NOX4 enzyme activities (Figure 4E) and ROS production (Figure 4G, 4H) in ACHN cells, whereas, a significant decrease in NADPH levels (Figure 4D), NOX4 activity (Figure 4F) and ROS accumulation (Figure 4I, 4J) were associated with the knockdown of G6PD in comparison to the Non-silencer cells. These results show that G6PD could alter the redox status and promote ROS production partly by increasing NADPH levels and NOX4 activity in RCC cells.

### p-STAT3 is increased in response to G6PD-facilitated ROS accumulation

**Figure 5:**
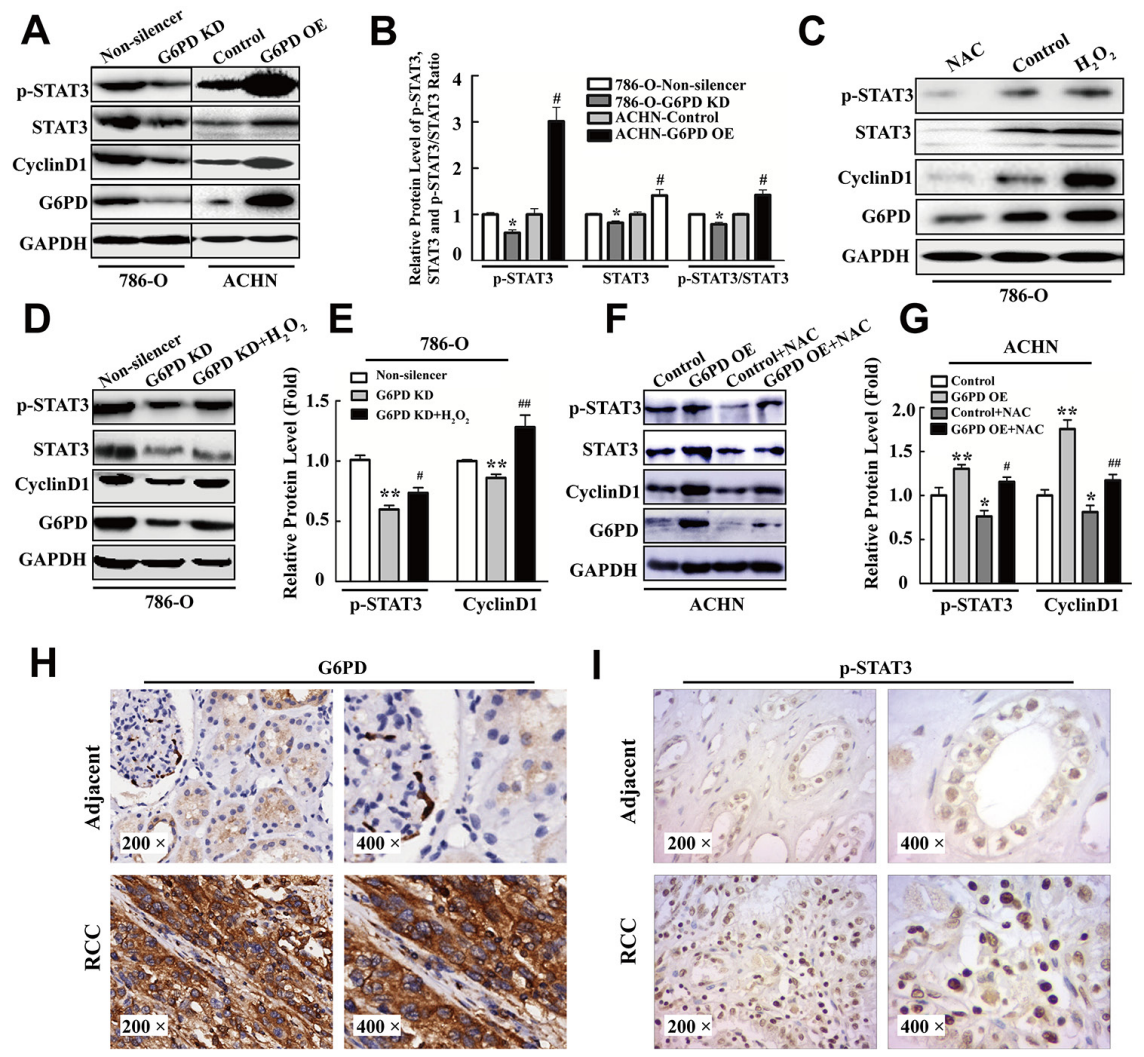
p-STAT3 is increased by G6PD-facilitated ROS accumulation in RCC **(A-B)** Western blot assay for p-STAT3, STAT3, CyclinD1 and G6PD expression level (A) and grayscale scanning analysis (B) in 786-O and ACHN cells with stable G6PD knockdown or overexpression. **(C)** 786-O cells were analyzed by Western blot for p-STAT3, STAT3, CyclinD1 and G6PD expression after treatment with 20 mM NAC for 24 h or 1 mM H_2_O_2_ for 2 h. Bars represent the means ± SD from three independent experiments, each performed in triplicate. ^*^ *p <* 0.05 vs. 786-O-Non-silencer; ^#^ *p <* 0.05 vs. ACHN-Control (unpaired Student *t*-test). **(D-G)** p-STAT3, CyclinD1 and G6PD expression levels were measured by Western blot (D, F) and grayscale scanning (E, G) after treatment with 1 mM H_2_O_2_ in 786-O cells for 2 h or 20 mM NAC in ACHN cells for 24 h. Bars represent the means ± SD from three independent experiments, each performed in triplicate. ^*^ *p* < 0.05, ^**^ *p* < 0.01 vs. Non-silencer or Control; ^#^ *p <* 0.05, ^##^ *p* < 0.01 vs. G6PD-KD or G6PD-OE (one-way ANOVA). **(H-I)** Immunohistochemistry analysis for G6PD (H) and p-STAT3 (I) were performed in adjacent noncancerous renal tissues (upper row) and RCC tissues (lower row). Representative images are shown (200× and 400×).

It has been reported that signal transducer and activator of transcription 3 (STAT3) plays a vital role in signal transduction pathways that mediate cell proliferation, partly by regulating CyclinD1 expression in RCC [25]. Furthermore, p-STAT3 (Ser727) has also been proved to be an independent prognostic factor for clear cell RCC [26]. These evidences lead us to suspect that p-STAT3 excessive activation may contribute to G6PD-stimulated RCC cell proliferation via up-regulated CyclinD1 expression. To test this hypothesis, Western blot analysis was firstly performed to identify the expression changes of p-STAT3 in RCC cells with G6PD overexpression or knockdown. The results showed that G6PD knockdown could notably attenuate the levels of p-STAT3, STAT3 and the ratio of p-STAT3/STAT3 in 786-O cells, whereas, with the G6PD overexpression, the protein level of both p-STAT3 and STAT3 were obviously increased in ACHN cells (Figure 5A, 5B). Meanwhile, the mRNA and protein levels of CyclinD1 were also down or up-regulated when G6PD was knockdown or overexpressed in RCC cells (Figure 5A, Supplementary Figure 2). These results indicate that G6PD may have the potential to increase p-STAT3 signaling activities and promote CyclinD1 transcription.

Our previous reports indicated that the aberrant ROS level could influence melanoma cell proliferation by regulating the DNA-binding activity of p-STAT3 [24]. In the present study, we next assessed whether the up-regulated expression levels of p-STAT3 were influenced by the effects of G6PD-facilitated ROS accumulation. The results showed that when 786-O cells were cultured with the ROS scavengers N-acetyl-cysteine (NAC) or H_2_O_2_ (used to evaluate the ROS generation), significant promotion or inhibition of ROS levels were observed in corresponding cells in comparison with the control (Supplementary Figure 3). Consistently, ROS accumulation further promoted the expression of p-STAT3, STAT3 and the ratio of p-STAT3/STAT3 in 786-O cells (Figure 5C, Supplementary Figure 4A). Significantly reduced or elevated CyclinD1 expression at both mRNA and protein levels was also observed when 786-O cell was treated with NAC or H_2_O_2_ (Figure 5C, Supplementary Figure 4B-4C). The above results imply that G6PD mediates p-STAT3 signaling activities, CyclinD1 overexpression and promoted RCC proliferation may be dependent on the up-regulation of cellular ROS accumulation.

To clarify this notion, we tested whether the ROS levels could influence the effects of G6PD dysregulation on p-STAT3 activation and CyclinD1 gene expression. The results of Supplementary Figure 5 showed that H_2_O_2_ addition could rescue the ROS down-regulation by G6PD knockdown in 786-O cells, whereas, NAC in ACHN cells could reverse the ROS up-regulation by G6PD overexpression. Moreover, the p-STAT3 and CyclinD1 expression reduction, mediated by G6PD knockdown, could be rescued by H_2_O_2_ addition in G6PD-knockdown 786-O cells (Figure 5D, 5E), while the up-regulated p-STAT3 signaling activities and CyclinD1 expression could be reversed by NAC stimulation in G6PD-overexpressing ACHN cells (Figure 5F, 5G). These results proved that G6PD might promote p-STAT3 activation by mediating ROS accumulation. We then analyzed the expression of G6PD and p-STAT3 in human RCC and paired adjacent non-cancer renal tissues (n=10) by immunohistochemistry. As shown in Figure 5H, 5I, the staining of both G6PD and p-STAT3 was stronger in RCC than in the adjacent tissues. Moreover, the Pearson correlation analysis revealed that the expression levels of G6PD protein are strongly correlated with p-STAT3 in human RCC samples (r = 0.521, *p* < 0.01). These results reveal that up-regulated co-expression of G6PD and p-STAT3 may synergistically contribute to the tumorigenesis of RCC.

Taken together, G6PD promotes tumor cell proliferation possibly through ROS-stimulated persistent activation of p-STAT3 signaling and up-regulated CyclinD1 expression in RCC.

### p-STAT3 binds directly to the G6PD promoter

p-STAT3 is a transcriptional factor that preferentially binds to the TTN5AA consensus regulatory sequences of its target genes and activates their expression [27, 28]. Interestingly, after the 786-O cells were treated with NAC or H_2_O_2_ as above, we could observe a significant decrease or increase of G6PD expression at both mRNA and protein levels (Supplementary Figure 4D-4E and Figure 5C), which was consistent with the p-STAT3 signaling activity changes. Therefore, we hypothesize that p-STAT3 may be a novel transcriptional regulator of G6PD gene expression, and thus form a positive feedback loop to contribute to G6PD up-regulation in RCC.

**Figure 6:**
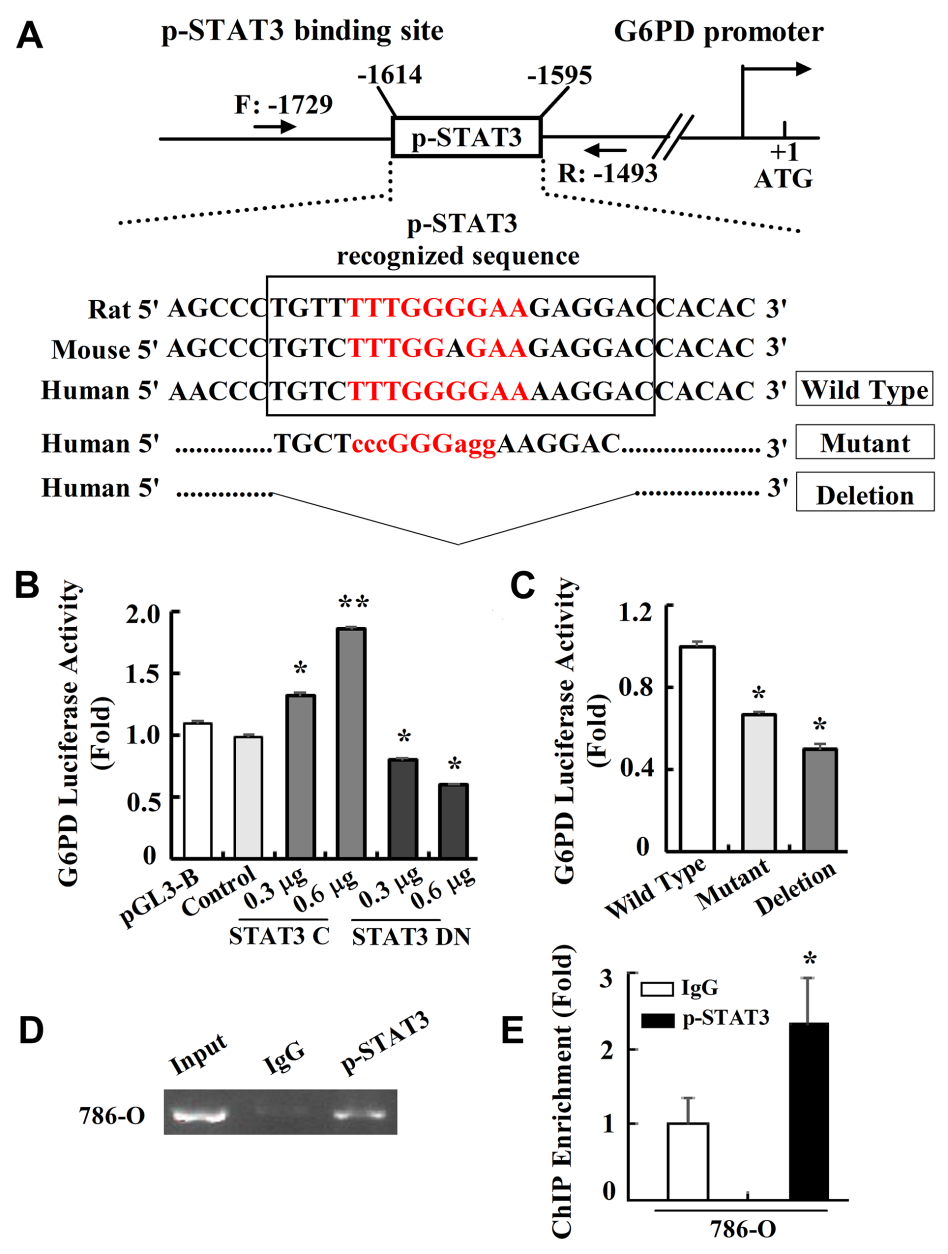
p-STAT3 binds directly to G6PD promoter **(A)** MatInspector database analysis of the consensus binding site for p-STAT3 on the G6PD promoter. Sequences containing mutant or deleted base pairs were used to establish the G6PD-luc mutant or deletion luciferase reporter constructs. **(B-C)** Luciferase reporter assays of p-STAT3 transcriptional activity on the wide type (B) or mutant (C) luciferase reporter constructs were performed. ^*^ *p* < 0.05, ^**^ *p* < 0.01 vs. Control or Wild type (one-way ANOVA). **(D-E)** ChIP assay was performed in 786-O cells by RCR (D) or real-time PCR (E) using anti-p-STAT3 antibody or normal IgG as a control. Data represent three independent experiments, each performed in triplicate. Bars represent the means ± SD. ^*^ *p* < 0.05 vs. IgG (unpaired Student *t*-test).

To investigate whether p-STAT3 activates G6PD transcription, bioinformatics analysis was performed using MatInspector software. The results showed that the transcriptional regulatory region of G6PD had conserved p-STAT3 binding TTN5AA sequences (Figure 6A). We then constructed G6PD-luc which contained the p-STAT3 binding site on the G6PD promoter for the luciferase reporters assay. Following the transfection of G6PD-luc and vectors encoding either the mutant STAT3 C or STAT3 DN, which actively up or down-regulates STAT3 signaling pathway persistently [28, 29], the stimulated increase or reduction of G6PD-luc activity occurred in a dose-dependent manner in 786-O cells (Figure 6B). The G6PD-luc mutant, or deletion containing the mutant or deleted p-STAT3 binding site (Figure 6A), exhibited significantly decreased p-STAT3 activity as compared to the wild type (Figure 6C), indicating that p-STAT3 plays an important role in activating G6PD mRNA expression.

To further assess whether p-STAT3 facilitates G6PD transcription by directly targeting the G6PD locus, a specific primer covering the potential p-STAT3 binding site located between -1729 and -1493 bp of the G6PD promoter was designed (Figure 6A). Using DNA fragments precipitated with anti-p-STAT3 antibody, the chromatin immunoprecipitation (ChIP) assay showed that p-STAT3 was recruited to the G6PD promoter about 2 folds as compared with IgG (Figure 6D, 6E), suggesting that p-STAT3 could bind on the G6PD transcriptional regulatory region. These data indicate that the functional p-STAT3 may act as a direct regulator of G6PD gene transcription in RCC cells.

### p-STAT3 contributes to G6PD overexpression in RCC cells

**Figure 7:**
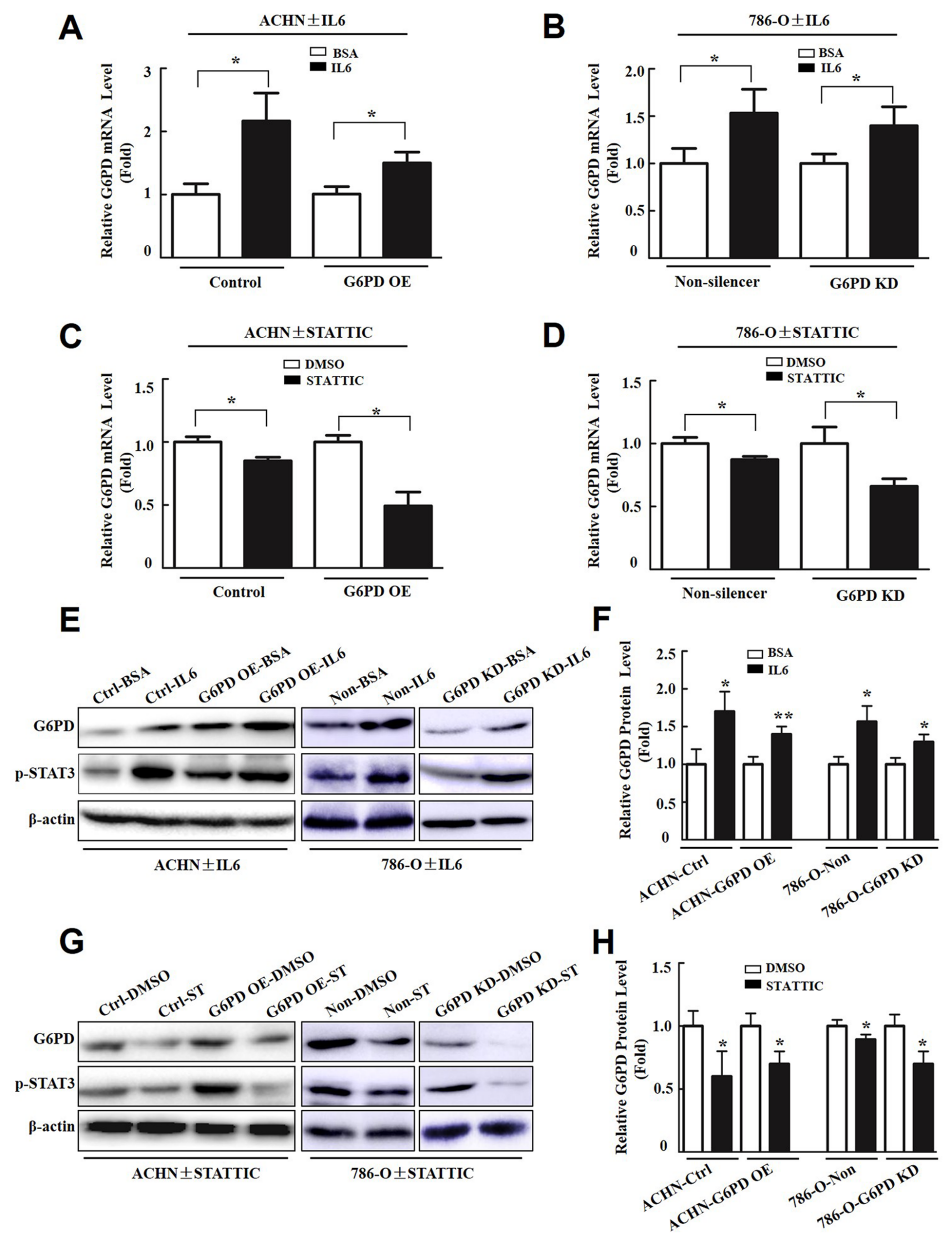
p-STAT3 contributes to G6PD overexpression in RCC cells Real-time RT-PCR **(A-D)**, Western blot **(E, G)** and grayscale scanning **(F, H)** show the expression changes of G6PD in ACHN and 786-O cells after treated with p-STAT3 activator (IL6, 4 ng/ml) or inhibitor (STATTIC, 6 μM) for 24 h. BSA or DMSO treated samples were regarded as “1 fold”. Each bar represents mean ± SD from three independent experiments, each performed in triplicate. ^*^ *p* < 0.05, ^**^ *p* < 0.01 vs. BSA or DMSO (unpaired Student *t*-test).

To explore whether p-STAT3 is involved in G6PD up-regulation in RCC, Real-time RT-PCR and Western blot were used to analyze the impact of p-STAT3 activators (interleukin 6, IL6) or inhibitors (STATTIC) on G6PD expression. The results showed that IL6, which promoted p-STAT3 activity, increased the levels of G6PD transcription in ACHN and 786-O stable cells by about 1.5∼2 folds relative to the BSA controls (Figure 7A, 7B). Whereas, the expression of G6PD at the mRNA level was notably reduced by the p-STAT3 signaling inhibitor STATTIC in both ACHN and 786-O cells (Figure 7C, 7D). Additional Western blot and grayscale scanning analyses showed that the significantly increased G6PD expression at the protein level was positively associated with the activation of p-STAT3 after treatment with IL6 (Figure 7E, 7F). Conversely, after stimulated with STATTIC, the expression of G6PD obviously decreased in RCC cells (Figure 7G, 7H). Taken together, these results show that persistent activation of p-STAT3 could act as a positive feedback regulator and lead to the aberrant transcription of G6PD in RCC.

## DISCUSSION

In the present study, we aimed to clarify the function of G6PD in promoting RCC proliferation and unravel the potential mechanisms underlying this regulation. Several studies have demonstrated that served as the main producer of ROS in RCC, NOX4 suppression could abrogate RCC cells proliferation, invasion, colony formation, and tumor growth [19, 20]. However, whether the oncogenic function of NOX4 is implicated with ROS generation remains largely undefined. Moreover, why NOX4 activities become up-regulated and the remaining specific mechanisms underlying the manipulation of ROS production in RCC still need to be elucidated. ROS have been implicated in cancer development through regulation of the redox state of target cells and exert diverse effects on cellular function, by promoting either cell proliferation and tumor progression, or cell death and tumor regression [30, 31], suggesting that ROS increment is a double-edged sword in tumor cells. Typically, cancer cells are under higher intrinsic levels of oxidative stress than normal cells. This phenomenon can be explained, at least in part, by the hyperactive metabolism reprograming taking place in malignant cells that is required for their rapid growth [32]. However, the levels and functions of ROS, as well as its regulatory factors in RCC development and progression, are largely unknown. Our present report unravel that G6PD plays a critical role in the regulation of RCC redox homeostasis and the elevated levels of NADPH might be an indispensable mediator of NOX4-regulated ROS accumulation. Inducting the exogenous G6PD overexpression up-regulates the production of NADPH, as well as the enzymatic activity of NOX4, and thus leads to the increment of G6PD-NADPH-NOX4-dependent ROS generation in the G6PD promoted RCC carcinogenesis. However, several problems, such as the gene expression alternations of NOX4 or other relevant regulators, remain to be clarified in the future researches.

The function of NOX-derived ROS in the kidney involves not only the alteration of cell fate, but also regulation of gene expression [15]. Pretto F *et al* found that RCC patients, showing over-activation of STAT3, frequently develop the cachectic syndrome, and that the STAT3 inhibitors are well worth evaluating as a therapeutic option for RCC patients [33]. Reducing p-STAT3 expression can not only significantly inhibit tumor cells proliferation [34, 35], but also migration and invasion in RCC [36]. The importance of ROS-STAT3 in cancer development and therapy has been well reported [37, 38]. Most studies suggested that ROS facilitated abnormal activation of the STAT3 signaling results from increased levels of STAT3 phosphorylation, rather than total STAT3 expression [37, 38]. Meanwhile, some other reports pointed that ROS affected total STAT3 expression via regulating Sp family transcription factors (TFs), demonstrating that STAT3 is a Sp-regulated target gene [39]. However, as a double-edged sword in tumor proliferation, malignant transformation, apoptosis suppression and immune evasion, ROS from different sources may have different or even completely opposite functions [1]. Here we demonstrate that STAT3, and its active form p-STAT3, are significantly promoted by G6PD-triggered increased ROS production. As a direct transcriptional regulator, STAT3 is constitutively activated and serves as an independent prognostic indicator in RCC [26, 40, 41]. Aberrant p-STAT3 directly binds to the promoter and regulates the expression of a series of cell-cycle genes including CyclinD1 that mediates cell survival, proliferation and chemotherapeutic sensitivity [42]. The high frequency of CyclinD1 overexpression observed in RCC patients suggesting that CyclinD1 might contribute to the tumorigenesis and aberrations in the G_1_/S transition of the cell cycle [43, 44]. Li S *et al* reported that p-STAT3 inhibition reduces the expression of STAT3-regulated cell survival, proliferation, and angiogenic factors including CyclinD1 [25]. Our data suggest that the CyclinD1 gene expression is positively correlated with the levels of p-STAT3, ROS accumulation and G6PD expression. Moreover, G6PD could regulate CyclinD1 but had a minimal effect on CDK2 expression in regulating cell cycle distribution of RCC cells (data not shown), implying that CyclinD1 must be a more potential downstream target of G6PD. Although further investigations are required to elucidate the mechanism involved in this regulation, our results definitely support the oncogenic role of G6PD in promoting RCC proliferation.

In this study, the biological functions and underlying molecular mechanisms of G6PD overexpression in RCC tumorigenesis have partially been clarified. However, the question why G6PD is highly expressed and exhibits aberrant activities in a number of human cancers is far from being answered. Previous studies have revealed that G6PD over-activation in human tumors may be attributed to the tumor suppressor p53, the most frequently mutated gene in human tumors, which binds to G6PD, inhibits formation of the active G6PD dimer, and suppresses NADPH production, as well as glucose consumption and biosynthesis [45]. In RCC cells, we find that both the expression level and the activity of G6PD enzyme are up-regulated. Nevertheless, given the fact that only 4% of ccRCCs present with p53 mutations, enhanced G6PD activities to direct glucose toward biosynthesis and increased tumor cells growth may not be due to p53 inactivation in RCC [46]. Liu B *et al* reported that G6PD is highly expressed in chronic hepatitis B virus (HBV)-associated liver cancers and up-regulated by HBx-mediated activation of Nrf2, which may be of importance in the reprogramming of glucose metabolism and development of hepatocarcinoma [47]. However, in RCC, the reason for G6PD up-regulation has not yet been reported. In this study, we attempted to explain the regulatory mechanism underlying G6PD overexpression. The results show that p-STAT3 could recruit to the transcriptional region of the G6PD promoter and activate its expression, demonstrating p-STAT3 has a positive feedback regulation in G6PD overexpression and sheds new light on the mechanism underlying G6PD dysregulation and clinical RCC carcinogenesis.

Both migration and invasion are important aspects in metastasis, with a high likelihood of poor prognosis. In this report, we find that G6PD expression in metastatic RCC patients is significantly higher than that of RCC specimens without lymph node or distant metastasis, indicating that G6PD might play an important role in mediating the progression of RCC to metastasis. Although the mechanism of G6PD in RCC is not fully clarified, we believe that in addition to the proliferation-promoting effect, G6PD may have other functions, especially the potential to facilitate migration and invasion of RCC. Therefore, more investigations are necessary to be carried out for testifying these hypothesis.

In conclusion, our findings demonstrate that G6PD is highly expressed in RCC. Aberrant G6PD could stimulate cell proliferation and tumor growth by activating the G6PD-ROS-p-STAT3-CyclinD1 signaling pathway. Moreover, p-STAT3 forms a positive feedback regulatory circuitry and contributes to G6PD dysregulation in RCC. Taken together, our research supports the oncogenic role of G6PD in human tumorigenesis, unveils new mechanisms underlying RCC carcinogenesis, and points to strategies of designing innovative therapeutic agents to improve RCC treatment.

## MATERIALS AND METHODS

### Transcriptomics data set analysis

To identify the expression profile of G6PD in human RCC, data mining was performed using the Gene Expression Omnibus (GEO, National Center Biotechnology information, Bethesda, MD, USA). One dataset, GSE53757 (http://www.ncbi.nlm.nih.gov/geo/query/acc.cgi?acc=GSE53757) containing 72 ccRCC (clear cell renal cell carcinoma, which accounts for 80∼90 % of all RCC cases) [7] and the corresponding control (normal or non-tumor) tissues was analyzed.

### Immunohistochemistry

Human RCC and renal tissue specimens were obtained from the department of pathology at the First and the Second Affiliated Hospital of Kunming Medical University, with the informed consent and approval from the Research Ethics Committee of Kunming Medical University. All specimens were subjected to immunohistochemistry analysis using the 2-step plus® poly-HRP anti-mouse/rabbit IgG detection system (PV-9000, ZSGB-BIO, Beijing, China) with anti-phospho-Stat3 (Ser 727) antibody (ab30647, Abcam, Cambridge, U.K.) or anti-G6PD antibody (ab133525, Abcam). The experimental procedure and immunohistologic analysis were performed as described before [4, 48].

### Cell culture and stable cell construction

The RCC cell lines ACHN (ATCC CRL-1611™), 786-O (ATCC CRL-1932™), Caki-1 (ATCC CRL-1611™) and the normal renal tubular epithelial cell line HK-2 (ATCC CRL-2190™) were purchased from Kunming Institute of Zoology (Chinese Academy of Sciences, China) and routinely cultured in MEM (#10370-021, Gibco™ Life Technologies, Grand Island, NY), RPMI-1640 (#11875-085, Gibco), McCOY’s 5A media (M9309, Sigma-Aldrich, Louis, MO, USA) and K-SFM medium (#17005-042, Gibco) containing 10% FBS, respectively.

To construct stable G6PD-overexpressing cell lines, 2 × 10^5^ ACHN or 786-O cells were plated in a 60-mm culture dish. At about 50% confluence, the cells were transfected with 2 μg pBABE-puro-G6PD (G6PD OE) or pBABE-puro (Control) plasmid as the control using Lipofectamine 2000 (#11668019, Invitrogen, Carlsbad, CA, USA). After 48h transfection, puromycin resistance screening (0.5 μg/ml) was performed. Single colonies were picked up at 21 days after transfection. To establish stable G6PD knockdown cell lines, the 786-O cells were transfected with pSR-GFP/Neo-G6PD shRNA (G6PD KD) or pSR-GFP/Neo-Non-silencer (Non-silencer) plasmid as the control and then selected using G418 resistance screening (1000 μg/ml) for 21 days. Single colonies were picked up and verified.

### Cell treatment, cell proliferation and cell cycle assays

STATTIC and H_2_O_2_ (sc-202818 and sc-203336) were purchased from Santa Cruz Biotechnology (SantaCruz, CA, USA). N-acetyl-cysteine (NAC, A7250) used as the ROS scavenger, was purchased from Sigma (Louis, MO, USA). STATTIC and NAC were dissolved in 100% dimethyl sulfoxide to prepare a 40 mM and 600 mM stock, respectively, and stored at −20°C. Recombinant human interleukin (IL)-6 purchased from R&D Systems (206IL, Minneapolis, MN, USA) was reconstituted in sterile PBS containing 0.1% bovine serum albumin to prepare a 10 μg/ml stock and stored at −20°C. The stock solution was added to the culture medium to achieve the indicated final concentrations. Cell proliferation was measured using the Cell Counting Kit-8 (CCK-8) (Dojindo Laboratories, Kumamoto, Japan), according to the manufacturer’s protocol. The plate clone formation assay, cell cycle assessment and data analysis were carried out based on our previous report [48–50].

### Animal experiments

All animal experiments were approved by the Institutional Animal Care and Use Committee of Kunming Medical University. 1 × 10^7^ RCC cells were resuspended in 200 μl PBS and injected subcutaneously into the right oxter flank of each six-week-old female BALB/c nude mice (purchase from Beijing HFK Bioscience Co., Ltd, Beijing, China), four mice per group. Tumor sizes were measured at indicated days, and tumor volumes were calculated according to the equation: volume = length × width^2^ ×(1/2). Following, the mice were sacrificed by euthanasia and the tumors were harvested.

### Real-time RT-PCR

Total RNA was isolated using the Trizol Reagent (Invitrogen™, Shanghai, China) based on the manufacturer′s instructions. Real-time RT-PCR amplifications were performed as described before [51] using different primers: G6PD: F: 5′-TGAGCCAGAT AGGCTGGAA-3′, R: 5′-TAACGCAGGCGATGTTGTC-3′; CyclinD1: F: 5′-GCGTACCCTGACACCCCTCTC-3′, R: 5′-CTCCTCTTCGCCTGATCC-3′; GAPDH: F: 5′-CGACCACTTTGTCAAGCTCA-3′, R: 5′-AGGGGTCTACATGGCAACTG-3′.

### Western blot

Cell lysates were prepared using the RIPA lysis buffer (R0010, Solarbio, Beijing, China) containing protease inhibitors (B14001, biotool, Shanghai, China). Western blot analysis was carried out as described before [52]. The antibody used in the experiment included the anti-G6PD antibody (ab133525, Abcam, Cambridge, U.K.); anti-phospho-Stat3 antibody (Ser 727) (ab30647, Abcam), and anti-CyclinD1 antibody (ab16663, Abcam). Additionally, anti-STAT3 antibody (#4904), anti-β-actin (#4967) antibody, and anti-GAPDH antibody (#2118) were purchased from Cell Signaling Technology (Beverly, MA, USA).

### G6PD and NOX oxidases activities

G6PD and NOX4 activities were analyzed using the G6PD or NOX4 assay kit (GMS70013.1 and GMS50096.1, GENMED, Shanghai, China) according to the manufacturer instructions. For the G6PD activity assay, 4 × 10^6^ cells were firstly pelleted using Reagent A. The cells were then lysed with 500 μl Reagent B in a microfuge tube and kept on ice for 30 min. Following, they were centrifuged at 13,000 × g for 5 min, and the supernatant was collected. 195 μl Reagent C, 25 μl Reagent D, and 25 μl Reagent E (5 μl Reagent F for NOX oxidases activity assay) were successively added into a labeled 96-well plate in duplicate, and the template was incubated in a 30°C incubator for 3 min. 5 μl of each sample (20 μg protein) or negative control (Reagent F) was pipetted into each well. For the NOX oxidases activity assay, 25 μl of cell sample (20 μg protein) or negative control (Reagent E) was transferred into each well. The absorbance was measured at OD 340 nm using the U-1800 ultraviolet spectrophotometer (Hitachi, Japan) at 0 min and 15 min. The activities of G6PD or NOX4 were then calculated according to the formula: Activity (U/mg) = (OD_sample_ - OD_negative control_ × 0.25 × dilution times) / 0.005 × 6.25 × 0.6 × 5.

### NADPH Levels

To measure NADPH levels, a colorimetric assay kit (k347-100, Bio Vision, Milpitas, USA) was used as described in the manufacturer instructions. 4 × 10^6^ cells were firstly pelleted for each assay in a microcentrifuge tube at 2000 rpm for 5 min. The cells were then lysed with 800 μl of NADP/NADPH Extraction Buffer in a microfuge tube and keep on ice for 10 min, followed by spinning down at 12,000 rpm for 10 min, and supernatant collection. Then, the extracted NADP/NADPH solution was transferred into a new labeled tube. To detect NADPH only, 200 μl were aliquoted into EP tubes. The samples were then heated to 60°C for 30 min in a water bath. Under the conditions, all NADP will decompose while NADPH will remain intact. The samples were then cooled on ice and quick-spun if precipitation occurred. 50 μl of NADPH samples were then transferred into a labeled 96-well plate in duplicates. 10 μl NADPH developer was added into each well and the reaction developed for 1h ∼ 4h. The plate was read at OD 450 nm in an U-1800 ultraviolet spectrophotometer (Hitachi, Japan). The OD 450 nm reading for each sample was compared to the standard curve and the amount of NADPH was expressed in μmol/μg protein.

### ROS level accumulation assays

Reactive oxygen species (ROS) in RCC cells were detected using the ROS Fluorescent Probe-DHE (R001, Vigorous Biotechnology, Beijing, China) and according to the supplier instructions [24]. Before the absorbance was assessed by flow cytometry, the images were firstly acquired with a BX51 fluorescence microscope (Olympus, Japan). Following, the fluorescence intensities were detected by flow cytometry at 530 nm excitation and 620 nm emission wavelengths.

### Luciferase reporter assays

The plasmid transfection and luciferase activity detection were performed as described before [51]. G6PD-luc, G6PD-luc Mutant and Deletion were commercially constructed by Shanghai Generay Biotech Co., Ltd (Shanghai, China). STAT3 C and STAT3 DN were purchased from Addgene (#24983 and #24984, Addgene, MA, USA).

### Chromatin immunoprecipitation (ChIP) assays

The ChIP assays were performed in 786-O cells according to the method described by Shang Y *et al* [53] using anti-phospho-Stat3 (Ser 727) antibody (ab30647, Abcam, Cambridge, U.K.) and primer (covering -1729 to -1493 of G6PD promoter region): F: 5′-TTGGCCAGTGCTCCAGAA-3′, R: 5′-ACCGCGAAACTGGGACTTTCT-3′.

### Statistical analysis

Bioinformatics analysis of the differences in G6PD expression between the ccRCC and control tissues were evaluated using the Wilcoxon rank-sum test in the R (http://www.r-project.org/) software environment. Bonferroni correction of the R function “p. adjust” was used to adjust the *p* values. Experimental analyses were carried out using SPSS version 20.0. For immunohistologic analysis, the differences of G6PD expression between RCC and paired adjacent normal tissues was assessed using the *χ*^2^ test; the differences of G6PD in adjacent normal tissues or RCC specimens from patients, with or without metastasis, were calculated using the Kruskal-Wallis one-way analysis of variances. The correlation between G6PD and p-STAT3 expression was assessed using Spearman correlation analysis. During animal experiments, the significance of the difference in tumor volumes between the groups was determined using two-way ANOVA. For the other analyses, the differences between groups were determined using unpaired Student *t*-test (n=2) or one-way ANOVA (n≥3). The data are expressed as means ± standard deviation (S.D.) of three independent experiments, each performed in triplicate. *p* < 0.05 was considered to be statistically significant.

## SUPPLEMENTARY MATERIALS FIGURES



## Abbreviations

G6PD: glucose-6-phosphate dehydrogenase
PPP: pentose phosphate pathway
RCC: renal cell carcinoma
ccRCC: clear cell renal cell carcinoma
ROS: reactive oxygen species
NADPH: nicotinamide adenine dinucleotide phosphate
NOX: NADPH oxidase
GEO: Gene Expression Omnibus
TCGA: The Cancer Genome Atlas
pRCC: papillary renal cell carcinoma
chRCC: chromophobe renal cell carcinoma
CCK-8: Cell Counting Kit-8
STAT3: signal transducer and activator of transcription 3
NAC: N-acetyl-cysteine
ChIP: chromatin immunoprecipitation
IL6: interleukin 6
HBV: hepatitis B virus
