# Supplementary material for: G6PD promotes renal cell carcinoma proliferation through positive feedback regulation of p-STAT3
Source: Oncotarget. 2017 Nov 20;8(65):109043–60. doi: 10.18632/oncotarget.22566 (PMC5752502; doi:10.18632/oncotarget.22566)
Supplement: Supplementary file 1 [file oncotarget-08-109043-s001.pdf]

G6PD promotes renal cell carcinoma proliferation through positive feedback regulation of p-STAT3

SUPPLEMENTARY MATERIALS

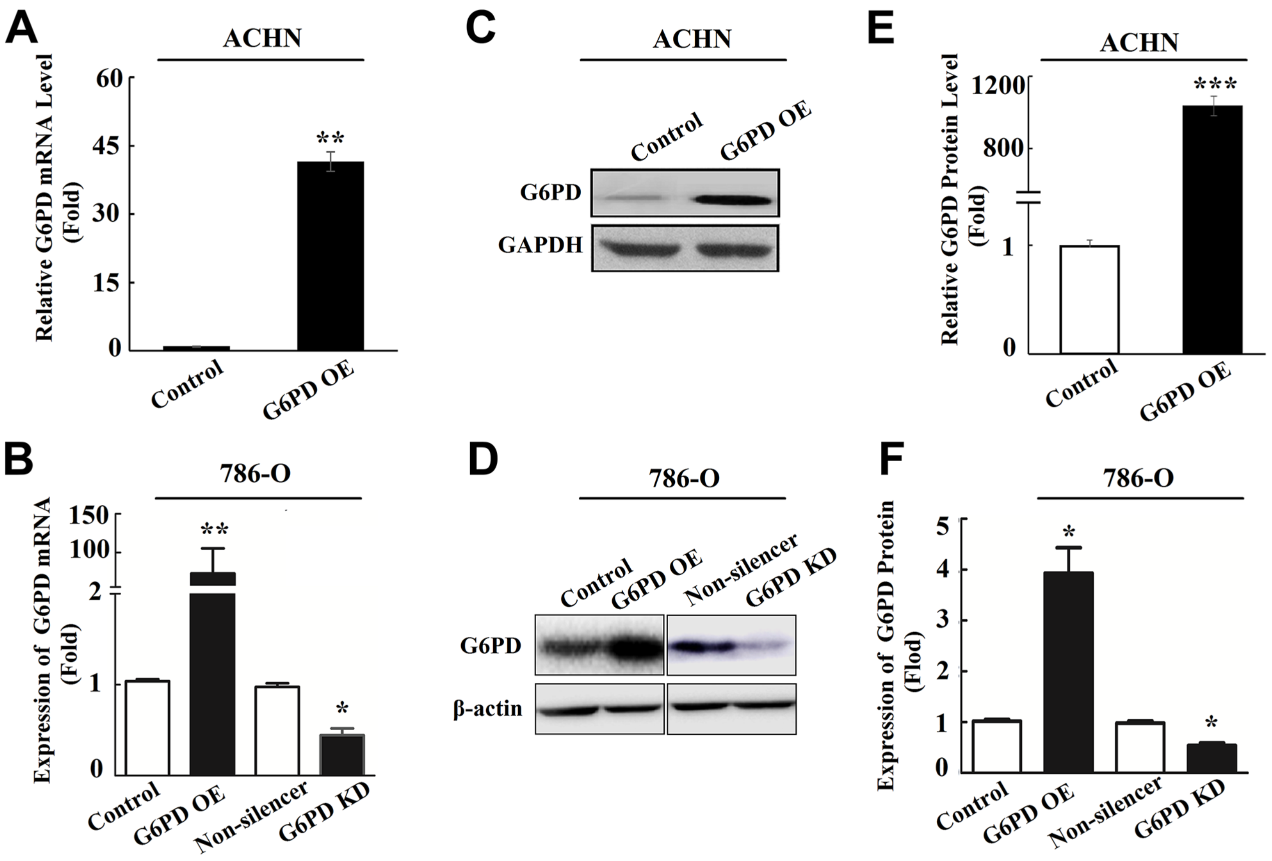

Supplementary Figure 1: Expression of G6PD in stably transfected RCC cells is confirmed by real-time RT-PCR and Western blot analysis. Real-time RT-PCR **(A-B)**, Western blot **(C-D)** and grayscale scanning **(E-F)** verification of G6PD levels in RCC cell lines with stable G6PD overexpression or knockdown by transfection with pBABE-puro (Control), pBABE-puro-G6PD (G6PD OE), pSR-GFP/Neo-Non-silencer (Non-silencer) or pSR-GFP/Neo-G6PD shRNA (G6PD KD) plasmid, respectively. Representative real-time RT-PCR and Western blots of three experiments are shown as A-B and C-D. GAPDH or β-actin served as an internal control. Grayscale scanning (E-F) was performed on three independent Western blot results for quantitative analysis. Bars represent the means ± SD. $^{*}p < 0.05$, $^{**}p < 0.01$, $^{***}p < 0.001$ vs. Control or Non-silencer (unpaired Student *t*-test).

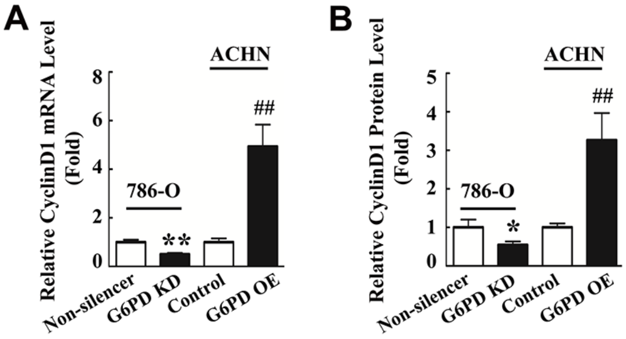

Supplementary Figure 2: mRNA and protein expression levels of CyclinD1 in stably transfected RCC cells. Real time RT-PCR assays **(A)** and Western blot analysis (**B**, grayscale scanning results) were performed in RCC cell lines with stable G6PD knockdown or overexpression. The data represent three independent experiments, each performed in triplicate. Each bar represents mean ± SD. ** $p <$ 0.01, $^{\#\#}$ $p < 0.01$, * $p < 0.05$ vs. Non-silencer or Control (unpaired Student *t*-test).

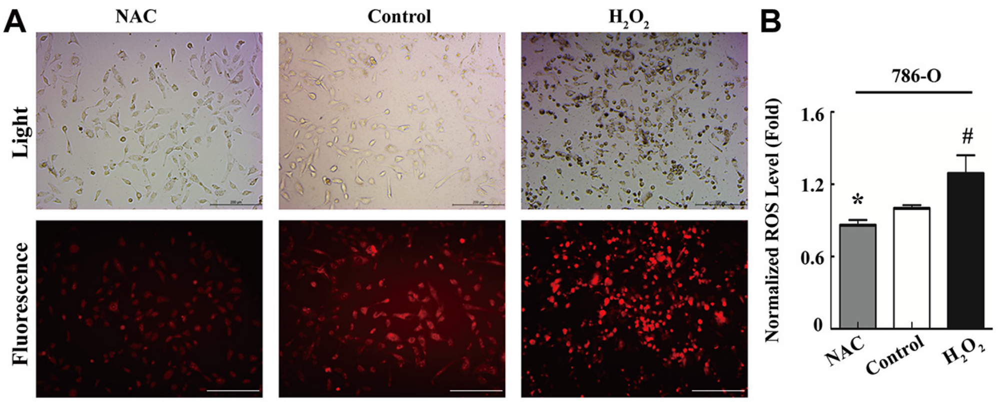

Supplementary Figure 3: ROS level detection in 786-O cells. Levels of ROS accumulation were measured by fluorescence microscope **(A)** and flow cytometry analysis **(B)** after 786-O cells treated with 20 mM NAC for 24 h or 1 mM H_2O_2 for 2 h. The upper images were taken under normal light and the bottom images were taken under fluorescence. Scale bar = 200 μm. Each bar represents mean ± SD from three independent experiments, each performed in triplicate. * $p < 0.05$, $^{\#}$ $p < 0.05$ vs. Control (one-way ANOVA).

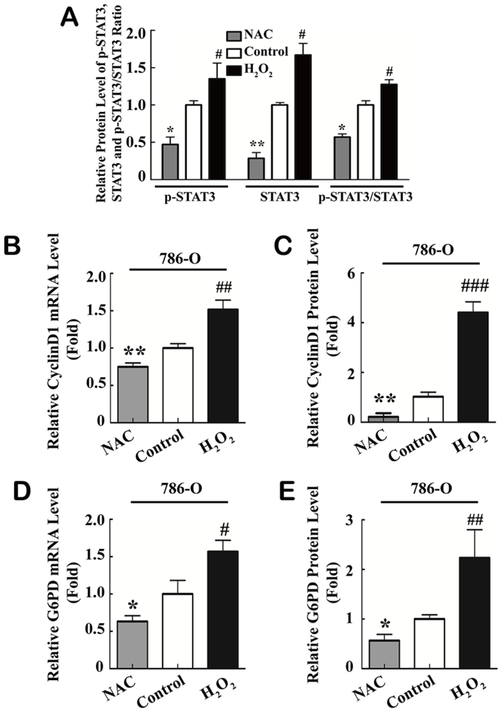

Supplementary Figure 4: Expression of p-STAT3, STAT3, p-STAT3/STAT3, CyclinD1 and G6PD levels were measured. Western blot analysis (**A, C, E**, grayscale scanning results) and Real time RT-PCR assays **(B, D)** were performed in 786-O cells after treated with 20 mM NAC for 24 h or 1 mM H_2O_2 for 2 h. The data represent three independent experiments, each performed in triplicate. Each bar represents mean ± SD. * $p < 0.05$, $^{\#}$ $p < 0.05$, ** $p < 0.01$, $^{\#\#}$ $p < 0.01$ vs. Control (one-way ANOVA).

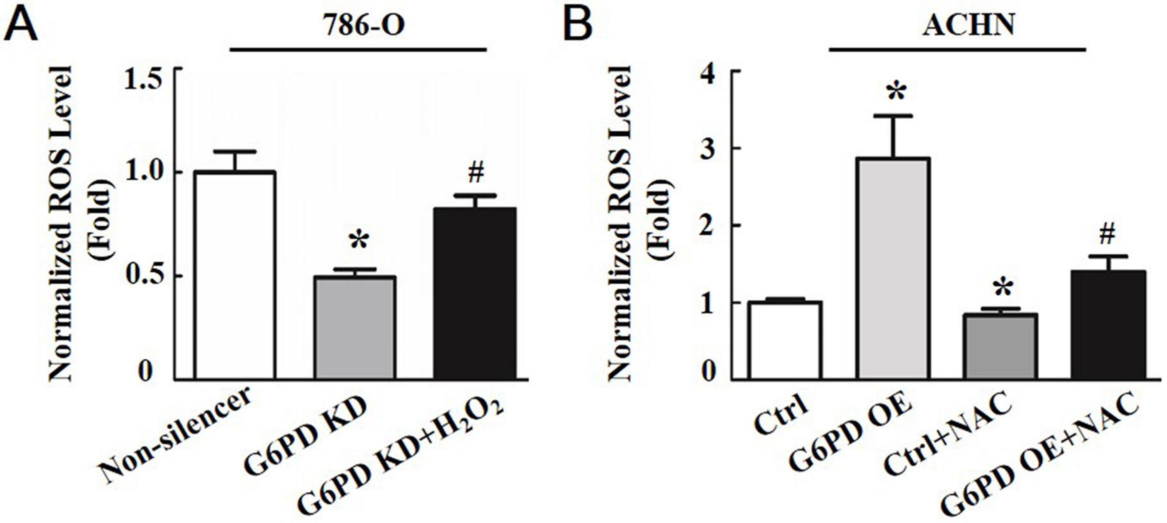

Supplementary Figure 5: Levels of ROS accumulation in RCC cells. Flow cytometry analysis for ROS level detection in 786-O **(A)** or ACHN cells **(B)** after treated with 1 mM H_2O_2 for 2 h or 20 mM NAC for 24 h, respectively. Each bar represents mean ± SD from three independent experiments, each performed in triplicate. * $p < 0.05$ vs. Non-silencer or Ctrl, $^{\#}$ $p < 0.05$ vs. G6PD KD or G6PD OE, respectively (one-way ANOVA).
